# Social conditions and disability related to the mortality of older people in rural South Africa

**DOI:** 10.1093/ije/dyu093

**Published:** 2014-05-15

**Authors:** F Xavier Gómez-Olivé, Margaret Thorogood, Philippe Bocquier, Paul Mee, Kathleen Kahn, Lisa Berkman, Stephen Tollman

**Affiliations:** ^1^School of Public Health, University of the Witwatersrand, Johannesburg, South Africa, ^2^INDEPTH Network (www.indepth-network.org), ^3^Warwick Medical School, University of Warwick, Coventry, UK, ^4^Centre de recherche en démographie et sociétés, Université Catholique de Louvain, Louvain-La-Neuve, Belgium, ^5^Centre for Global Health Research, Epidemiology and Global Health, Umeå University, Umeå, Sweden, ^6^Harvard Centre for Population and Development Studies and ^7^Harvard School of Public Health, Harvard University, Cambridge, MA, USA

**Keywords:** Mortality, ageing, HIV, disability, quality of life, South Africa

## Abstract

**Background:** South Africa is experiencing a health and social transition including an ageing population and an HIV epidemic. We report mortality experience of an older rural South African population.

**Methods:** Individual survey data and longer-term demographic data were used to describe factors associated with mortality. Individuals aged 50 years and over (*n* = 4085) answered a health and quality of life questionnaire in 2006 and were followed for 3 years thereafter. Additional vital events and socio-demographic data were extracted from the Agincourt Health and Demographic Surveillance System from 1993 to 2010, to provide longer-term trends in mortality. Cox regression analysis was used to determine factors related to survival.

**Results:** In 10 967 person-years of follow-up between August 2006 and August 2009, 377 deaths occurred. Women had lower mortality {hazard ratio [HR] 0.35 [95% confidence interval (CI) 0.28–0.45]}. Higher mortality was associated with being single [HR 1.48 (95% CI 1.16–1.88)], having lower household assets score [HR 1.79 (95% CI 1.28–2.51)], reporting greater disability [HR 2.40 (95% CI 1.68–3.42)] and poorer quality of life [HR 1.59 (95% CI 1.09–2.31)]. There was higher mortality in those aged under 69 as compared with those 70 to 79 years old. Census data and cause specific regression models confirmed that this was due to deaths from HIV/TB in the younger age group.

**Conclusions:** Mortality due to HIV/TB is increasing in men, and to some extent women, aged over 50. Policy makers and practitioners should consider the needs of this growing and often overlooked group.

**Key Messages**

- Lower quality of life (measured by WHOQOL), lower functionality (measured by WHODAS), lower household assets score and being single are associated with higher mortality levels in this older South African population.
- As in high-income countries, mortality in a poor rural setting presents with the ‘male-female survival paradox’ where women experience a higher number of risk factors than men but survive longer.
- Mortality due to HIV/TB, especially in men, has increased in recent years, so that mortality is higher in the younger old (up to 69 years of age), after which there is an apparent fall in mortality with increasing age.
- In South Africa, policy makers and practitioners should consider the needs of the expanding, but often overlooked, older population.

## Introduction

To date there has been limited effort to investigate the way the HIV epidemic, the growing burden of non-communicable disease, and social conditions and disability affect older people’s mortality in rural African settings.^1^^,^^2^ At the same time, there is limited knowledge of the health experience, quality of life and the factors associated with mortality in older people living in rural sub-Saharan Africa. The data reported in this paper were collected as part of the Study on Global Ageing and Adult Health (SAGE), which set out to improve knowledge on the health, disability and well-being of older populations in low- and middle-income countries.^3^ Adult mortality has been increasing in southern and eastern countries of sub-Saharan Africa since the 1990s, mainly as a result of the HIV epidemic.^4^^,^^5^ Most studies have focused on young adults, ignoring the fact that the older population is also directly and indirectly affected by the HIV epidemic.^6–8^ Existing data show that one effect of the HIV/AIDS epidemic is to increase the number of older people living alone.^7^ However, there has been limited attention in the literature to the prevalence of HIV in older people, despite calls to examine infection and mortality in the population aged 50 years and older.^1^^,^^9^ Negin *et al*. have called attention to the interplay between the ageing process and the double burden of HIV and non-communicable diseases, especially in Africa.^10^

This paper describes factors associated with mortality in people aged 50 years and over in a rural area of South Africa where HIV prevalence is high^11^ and where gender differences in experience of quality of life, function and health have been described.^12^ The objectives of this paper are twofold: first, to describe the mortality trends and identify possible HIV-related trends; and second, we aimed to identify the social and functional risks related to increased mortality risk in this cohort. In exploring the social and functional risks, we considered both the well-known and frequently described ‘distal causes of disease’^13^ including education, occupation^13^ and poverty,^14^ as well as other factors which have been previously associated with higher mortality risk in this or similar populations. Union (partnership) status has been related with poor self-reported health, quality of life and well-being in older people in different African settings.^3^^,^^15^^,^^16^ A single question on self-rated health and the composite measure on functionality (WHO-DAS) have been associated with higher risk of mortality in India^17^ whereas, in Agincourt HDSS, former Mozambican refugees have been found to suffer higher levels of child mortality.^18^ To further explore the patterns of mortality, we have included information from the site-specific annual census from 1993 to 2010.

## Methods

Ethical clearance for the MRC/WITS Rural Public Health and Health Transitions Research Unit (Agincourt) and its associated Health and Demographic Surveillance System, and for the Agincourt-INDEPTH Study on Global Ageing and Adult Health, was granted by the Committee for Research on Human Subjects (Medical) University of the Witwatersrand, Johannesburg, South Africa, Refs M960720 and R14/49, respectively.

The study population and general methods have been described in detail previously.^12^ Here, we describe them briefly.

### Setting

In 1992, the MRC/Wits Rural Public Health and Health Transitions Research Unit initiated the Agincourt Health and Demographic Surveillance System (HDSS) in the rural Agincourt sub-district of Ehlanzeni district, Mpumalanga Province, South Africa. The total population under surveillance in 2006 was approximately 70 000 people distributed in 21 villages and 11 734 households. Despite substantial socioeconomic development in the area since then, infrastructure remains poor and formal unemployment rate is high (36%) with continuing high levels of labour migration among men and, increasingly, women.^19^ The public health system in the sub-district consists of six clinics and one health centre, and three district hospitals situated between 25 and 45 km from the study site.^20^ In 2008 a private health centre was built in one village, intended mainly for the treatment and care of people with HIV. HIV prevalence among all ages from 15 years in the area was 19.4% in 2010, with a peak of 45.3% for males and 46.1% for females at ages 35 to 39. The population over 50 years of age had an HIV prevalence of 16.5% (17.7% for males and 16.1% for females).^11^

Basic demographic data (pregnancy outcome, deaths, migration) are collected every year by trained local fieldworkers, to update HDSS census information.^21^ Additional individual and household data (food security, labour participation, household assets) are collected at regular intervals to provide contextual information. Although the surveillance system includes information on all deaths, there is incomplete official death notification and very few people can report their relatives' cause of death. To determine cause of death, we use two independent clinician assessments of verbal autopsy interviews. Verbal autopsies are carried out by trained lay fieldworkers with closest caregivers of the deceased for every death in the area, allowing us to study mortality by likely cause of death.^22^

#### Sample

In August 2006, there were 8429 people aged 50 years or over enumerated in the Agincourt HDSS. Of those, we excluded 575 who had been randomly selected to participate in a previous recent study; and those who were temporary (often labour) migrants (*n* = 2223), living for less than 6 months of the year prior to the study in the sub-district. The remaining 5631 individuals, who had been permanently resident in the area for 12 months prior to the 2006 census round, were invited to participate. Each person was visited a maximum of three times to attempt to complete the interview. Due to the census field constraints, we were not able to follow up any further those individuals who were absent from their households on these three visits. As a result, 1616 people (28.7%) were not interviewed although they were apparently permanently living in the study site. A further 458 (8.1%) individuals had died or were too sick to answer the questions, and 47 (0.8%) declined to take part. A total of 4085 individuals (response rate of 72.5%) participated.^12^

#### Data collection

We used a data collection tool that was adapted from the World Health Organization (WHO) –INDEPTH Network Study on Global Ageing and Adult Health (SAGE)^23^ to interview participants during 2006 census. The SAGE questionnaire included questions on self-rated health assessment, physical and cognitive function, well-being and quality of life. It was translated into the local language (Shangaan) and back-translated into English.

Additional data on gender, age, whether married or living with a partner, nationality, level of education and date and cause of death were extracted from the Agincourt HDSS (2006 census round). Employment status was indicated by being in paid work or not.

We also used HDSS census data, annually updated from 1993 to 2010, on population size and mortality to obtain the person-years of observation, and to determine out-migration and death dates.

### Follow-up

A total of 2006 study participants were followed up at annual census updates from 2007 to 2009, providing information on deaths up to 31 July 2009. Of the 4085 respondents, 38 (0.9%) were lost to follow-up with no information and have been excluded from analyses. The 4047 remaining participants were followed up until out-migration, death or 31 July 2009, whichever came first. Out-migration was defined as an individual moving away from his/her original dwelling. All efforts were made to follow up those who moved within the sub-district, but those we could not find in a new dwelling (48, 1.2%) and those who left the study area (56, 1.4%) were censored at out-migration date. Main reasons for out-migration were moving to new houses, change of union status, or work-related issues.

#### Variables

We calculated age at interview from recorded date of birth and reported age. We assigned participants to 5-year age groups.

Education was categorized according to the WHO levels of education: 6 years or more of formal education; less than 6 years of formal education; and no formal education. This information was obtained from the 2006 Agincourt HDSS database.

Since many unions are traditional rather than civic, and polygamy is practised by some people, we decided to categorize union status into two groups: currently married or living as married (in a current partnership); and single, including anyone not in a current partnership (i.e. those who had never married or were separated, divorced or widowed).

To evaluate socioeconomic status (SES), we used an ‘absolute SES’ indicator constructed from the 2005 household asset survey. Each asset was given equal weight by rescaling so that all values of a given asset variable fell within the range [0, 1]. Assets were categorized into five broad groups: ‘modern assets’, ‘power supply’, ‘water and sanitation’, ‘quality of housing’ and ‘livestock assets’. For each household and for each asset group, the asset values were added and then rescaled to arrive at a specific value in the range [0,1]. Finally, for each household these five group-specific scaled values were added to give an overall asset score with a value in the range [0, 5].^24^

Household asset scores were calculated using 2005 census data, since these were the most recent data available, and then grouped in quintiles for the entire Agincourt sub-district population. As participants in this study are a sub-sample of the whole population (50 years and older), they are not equally distributed across the five quintiles.

Due to the war in Mozambique, the Agincourt area received a high number of Mozambican refugees before 1993. Those who elected to remain in the area were recorded as Mozambican. The variable ‘nationality of origin’ refers to whether the participant was originally from South Africa or Mozambique. The Mozambican group are separately identified in the HDSS data and differ from the original South African population in measures such as education, household assets and child mortality.^18^ Many Mozambicans have now taken South African nationality which allows them to work legally and receive state pensions.

The physical and social functions of each participant were measured using the WHODAS II scale (World Health Organization Disability Assessment Schedule II) which assesses daily functioning.^25^ This scale is created by asking 12 questions on the difficulty experienced when performing certain activities in past 30 days. The score ranges from 0 to 100, with a high score indicating severe impairment of physical function. WHOQOL (Word Health Organization Quality of Life) was used to measure life satisfaction. WHOQOL includes questions on satisfaction with own health, personal and social relations, performing daily activities and overall satisfaction with life. It is presented on a scale of 8 to 40 (where 8 is the best quality of life).^26^^,^^27^ We used two single questions to measure self-rated health and working difficulty, where we asked the participants ‘In general, how would you rate your health today?’ with the options of very good, good, moderate, bad and very bad; and ‘Overall in the last 30 days, how much difficulty did you have with work or household activities?’ with options being none, mild, moderate, severe and extreme/cannot do.

### Data entry and analysis

We entered data using CSPro 3.1 data entry programme (http://www.census.gov/ipc/www/cspro/index.html) which includes validation checks. Data were then extracted to Stata10.1 (Stata Corp, College Station, TX, USA) for analysis.

#### Demographic analysis

A demographic analysis on the full Agincourt HDSS population from 1993 to 2010 was carried out to verify whether the sample mortality trend by age group was due to a sample bias or whether it is explained as a new mortality trend in the study site—that is, up to cohort recruitment. Hazard mortality ratios were computed using number of deaths that occurred within the HDSS as the numerator, and length of time lived in the HDSS over the period as the denominator, expressed in person-years. Individuals were considered residents when they spent more than 6 months per year in the HDSS area. In order to minimize effects on mortality of temporary migrants that return home to die,^28^ the mortality analysis did not included temporary migrants who died outside the HDSS or within 6 months of their return to the study site. The analysis of all-cause mortality was conducted by gender, 5-year age-group defined by age in August 2006 and 5-year time period. HIV/TB mortality was similarly analysed. This demographic analysis provided background mortality trends to compare with results from the participants in this study.

#### Cox regression model

We carried out a Cox’s regression analysis to determine the factors related to the risk of death. We first carried out an age-adjusted analysis of the impact of the three composite measures to evaluate whether gender effects could be merely explained by age differences (Table 2). Then we performed a univariate analysis (Table 3) and then constructed a multivariate model, including age group (treated as a categorical variable), sex, whether in a union, education status and household asset score as a-priori variables. We then entered in turn each of the other variables listed in Table 3, where the univariate analysis returned a *P*-value of less than 0.1. In each case, we compared models with and without the variable, and discarded the variable if the likelihood ratio test returned a value of *P* > 0.1. Three models, for the whole population, for men and for women, were constructed. Proportional analysis was performed using proportion hazard assumption. We performed a test of proportionality for each predictor using the Schoenfeld and scaled Schoenfeld residuals. If the tests were not significant (*P*-values > 0.05), proportionality was not rejected and we assumed that there was no violation of the proportional assumption. Once we had constructed the model for the whole population, we further investigated the effect of three groups of causes of death (chronic disease; HIV and TB; and other infections) by running the model separately for each of these cause groups.

## Results

There were 10 967 person-years of follow-up, with 377 deaths recorded. Participants were predominantly female (75.2%), which represents the demographics of this older population in the sub-district, with a mean age of 66.1 years at the time of interview. Less than half of the population had received any formal education. Men were more than twice as likely to be currently in a union. Most of the participants were retired and not looking for work, but 5.7% reported that they were unemployed and looking for employment.^12^ Most (56%) of the respondents in paid work were aged less than 60. A higher proportion of participants than expected, especially men, were living in households with no persons under 50 years of age (9.6%), or in a skip generation household (2.4%) where there were children (under 18 years) present but no adults aged under 50 years (Table 1).

**Table 1.** Demographic and health variables of persons 50 years old or older in the Agincourt sub-district cohort (2006–09) by gender

Variables	Male (*N*=1003, 24.8%)	Female (*N*=3044, 75.8%)	*P*-value (difference male vs female)
	***N* (%)**	***N* (%)**	
Mean age (95% CI)	67.8 (67.1–68.4)	66.1 (65.7–66.4)	<0.001
Age group (years)			
50–54	129 (12.9)	518 (17.0)	7 d.f. <0.001
55–59	143 (14.3)	495 (16.3)	
60–64	116 (11.6)	460 (15.1)	
65–69	204 (20.3)	436 (14.3)	
70–74	132 (13.2)	391 (12.8)	
75–79	135 (13.5)	410 (13.5)	
80–84	60 (6.0)	183 (6.0)	
85+	84 (8.4)	151 (5.0)	
Education status^a^			
Completed 6+ years of schooling	208 (20.7)	379 (12.5)	3 d.f. <0.001
Completed less than 6 years of schooling	210 (20.9)	539 (17.7)	
No formal education	545 (54.3)	2035 (66.9)	
Data missing	40 (4.0)	91 (3.0)	
Union status^b^			
In current partnership	768 (76.6)	1082 (35.6)	1 d.f. <0.001
Single	235 (23.4)	1962 (64.5)	
Living arrangement^c^			
Only adults aged 50 plus	158 (15.8)	230 (7.6)	2 d.f. <0.001
Skip generation	19 (1.9)	76 (2.5)	
Presence of adults younger than 50 years of age	826 (82.4)	2738 (90.0)	
Employment^d^			
Not working	756 (75.4)	2474 (81.3)	2 d.f. <0.001
Working	196 (19.5)	376 (12.3)	
Data missing	51 (5.1)	194 (6.4)	
Nationality of origin			
South African	761 (75.9)	2183 (71.7)	2 d.f. 0.037
Mozambican	240 (23.9)	854 (28.1)	
Data missing	2 (0.2)	7 (0.2)	
Household asset score^d^			
Highest	292 (29.1)	694 (22.8)	5 d.f. 0.001
High	199 (19.8)	664 (21.8)	
Medium	185 (18.4)	586 (19.3)	
Low	147 (14.7)	557 (18.3)	
Lowest	153 (15.3)	442 (14.5)	
Data missing	27 (2.7)	101 (3.3)	
Health status			
Highest	157 (15.7)	634 (20.8)	<0.001
High	168 (16.8)	593 (19.5)	
Medium	172 (17.2)	556 (18.3)	
Low	215 (21.4)	635 (20.9)	
Lowest	291 (29.0)	626 (20.6)	
WHODAS II^f^			
Best	327 (32.6)	697 (22.9)	<0.001
Good	181 (18.1)	536 (17.6)	
Medium	164 (16.4)	521 (17.1)	
Bad	157 (15.7)	637 (20.9)	
Worst	174 (17.4)	653 (21.5)	
WHOQOL^g^			
Lowest	207 (20.6)	593 (19.5)	<0.001
Low	168 (16.8)	673 (22.1)	
Medium	166 (16.6)	600 (19.7)	
High	217 (21.6)	616 (20.2)	
Highest	243 (24.2)	560 (18.4)	
Data missing	2 (0.2)	2 (0.1)	

^a^Number of completed years of education.

^b^In current partnership refers to those in a union; single includes never in a union, widowed, divorced or separated.

^c^Household structure was divided in three categories: (i) only adults 50 years or older; (ii) ‘skip generation’ (defined as a household were a person older than 50 lives with children under 18 years of age and there are no persons 18–49 years of age; (iii) ‘younger adults’ (defined as presence of both persons older and younger than 50 years in the household).

^d^Formally employed in 2004.

^e^Household asset score is a household weighted measure used as a proxy to calculate socioeconomic status.

^f^World Health Organization Disability Assessment Schedule II, a self-rated measure of functionality, presented in quintiles.

^g^World Health Organization Quality of Life is a self-rated measure of overall satisfaction with life, presented in quintiles.

We calculated death rates in 5-year age cohorts and found that in the period 2006–10, the male death rate increased steadily from age group 50–54 to age group 60–64 but then fell, only finally surpassing the 60–64 rates in the group over 85 years of age (see Supplementary Table 1, available as Supplementary data at *IJE* online). A similar but less marked pattern was observed in women. When we compared the study men and women death rates with the Agincourt census death rates for the periods 2003–06 and 2007–10, we only found a significant difference between the study [death rate 31.4/1000 (95% CI 17.4–56.8)] and the period 2003–06 [death rate 69.3/1000 (95% CI 57.6–83.3)] for men’s age group 50–54 years (see Supplementary Table 1, available as Supplementary data at *IJE* online).

**Figure 1.**
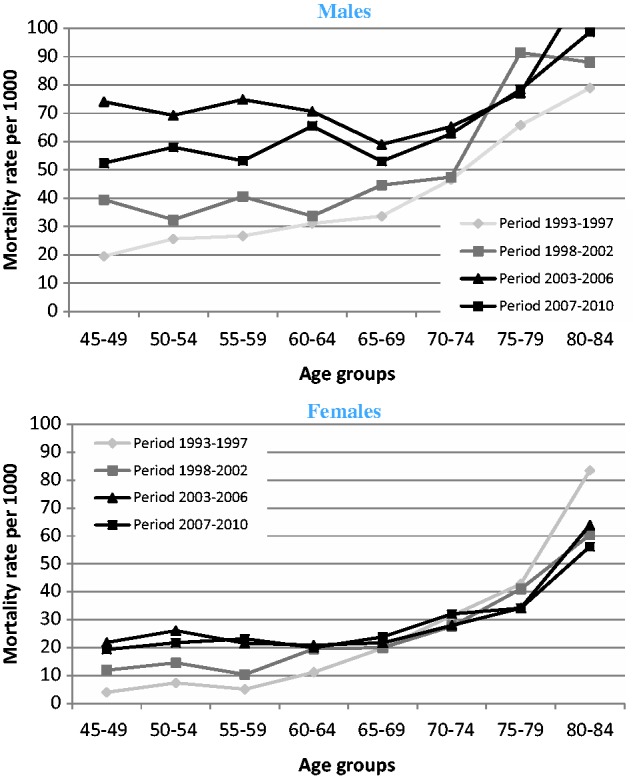
All-cause death rates by gender, age group and time period (1993 to 2010) in Agincourt sub-district (South Africa).

The relationship between age group and mortality led us to analyse mortality data over the entire period of the annual Agincourt census, from 1993. In Figure 1 we show the death rates per 1000 person-years for men and women by age group in four time periods from 1993 to 2010. Death rates in the younger men increased in each of the first three time periods up to 74/1000 in the 2003–06 period. This time period shows a drop in mortality for men aged 65–69 after an essentially flat line in the four younger age groups. In the final time period the death rates are lower, but with a similar pattern. Women showed a similar but less marked pattern.

**Figure 2.**
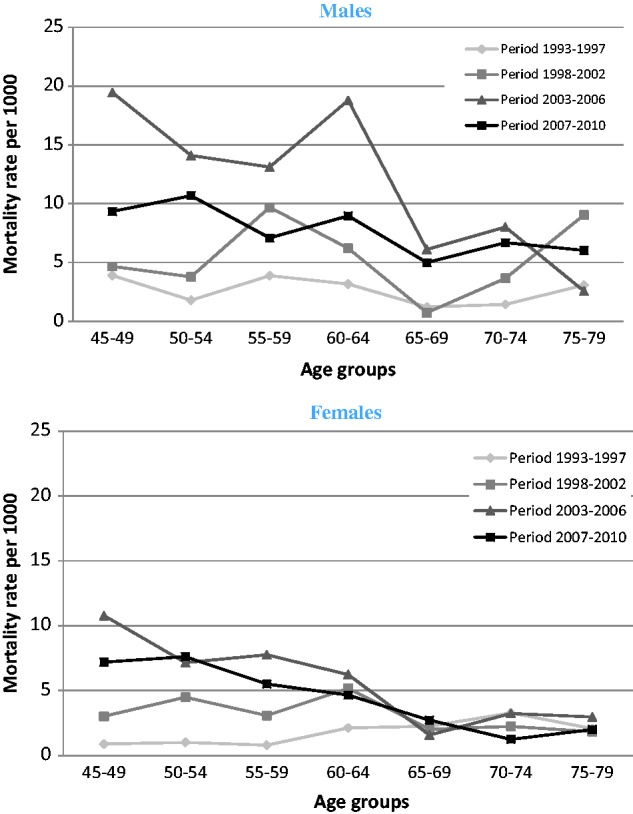
HIV/TB death rates by gender, age group and time period (1993 to 2010) in Agincourt sub-district (South Africa).

In Figure 2 we show similar graphs for the cause-specific death rates from HIV and TB, as determined by verbal autopsy. These rates rose markedly in the men aged under 65 years between the 1998–2002 period and the 2003–2006 period, but then fell in the later 2007–10 period. Again, women showed a similar but less marked pattern in HIV/TB death rate. When mortality contribution of HIV/TB is calculated as a percentage of all deaths,we observed that, in the peak period 2003–06 and in the youngest age group 45–49 years, HIV/TB contributes with 25% of the deaths in men and 50% of the deaths in women.

Because of this complex relationship between age, gender and risk of death, age could not be treated as a continuous variable with a linear relationship to survival. We therefore entered age into the Cox regression analysis as a categorical variable in 5-year age groups, allowing an independent estimation of risk for each age group.

The age-adjusted analysis of the three composite measurements, i.e. quality of life, functionality and health status, shows that gender effects are not the result of age differences for mortality or for composite health outcomes (Table 2).

**Table 2.** Age-adjusted Cox regression analysis of risk of death by composite measurement of persons 50 years old or older in the Agincourt sub-district cohort (2006–09) by gender

Score	Male	Female
	Hazard ratio (95% CI)	Hazard ratio (95% CI)
WHOQOL		
Lowest	1	1
Low	0.47 (0.29–0.77)	0.69 (0.50–0.98)
Medium	0.53 (0.33–0.85)	0.44 (0.29–0.67)
High	0.60 (0.39–0.92)	0.42 (0.27–0.64)
Highest	0.26 (0.15–0.45)	0.47 (0.31–0.72)
WHODAS		
Best	1	1
Good	1.51 (0.87–2.60)	0.76 (0.44–1.33)
Medium	1.46 (0.84–2.55)	1.28 (0.78–2.01)
Bad	2.38 (1.43–3.95)	1.46 (0.93–2.29)
Worst	3.48 (2.19–5.54)	2.57 (1.70–3.90)
Health status		
Lowest	1	1
Low	0.66 (0.42–1.03)	0.59 (0.40–0.85)
Medium	0.40 (0.24–0.67)	0.50 (0.33–0.76)
High	0.36 (0.22–0.59)	0.49 (0.33–0.73)
Highest	0.32 (0.20–0.51)	0.37 (0.24–0.58)

Table 3 shows the results for the univariate analysis of mortality risk for all demographic and health variables, and for the fully adjusted model. In the univariate analysis, women had lower mortality risks and, as previously described, there was a complex relationship with age. Not being in a current partnership, living in a household of lower socioeconomic status and having no formal education were all related to increased mortality. In general, older people living in households with younger adults and/or in ‘skip generation’ households have lower mortality. This protective effect remained when the analysis was carried out separately by gender, but only the effect of living with younger adults had confidence intervals below 1. People who reported that they were suffering moderate or bad health and those who reported severe difficulty with work or household duties experienced higher mortality, as did those who reported poor function, low quality of life and lowest health status.

**Table 3.** Univariate and multivariate Cox regression analysis of risk of death (2006–09) in study participants by gender

	Univariate analysis hazard ratios			Multivariate analysis hazard ratios		
	Total population	Male	Female	Total population	Male	Female
**Sex**						
Male	1			1		
Female	10.45^a^			0.35^a^		
Age group						
50–54	1	1	1	1	1	1
55–59	1.04	1.33	0.90	0.9	1.14	0.81
60–64	1.16	2.37^a^	0.73	1.11	2.10^a^	0.71
65–69	1.81^a^	2.02	1.50	1.44	2.03^a^	1.2
70–74	1.43	1.7	1.23	1.01	1.33	0.95
75–79	1.63^a^	1.57	1.59	1.06	1.23	1.06
80–84	2.04^a^	2.02	1.99^a^	1.30	1.26	1.37
85+	4.27^a^	4.11^a^	3.84^a^	2.27^a^	3.03^a^	2.07^a^
Education status^b^						
More than 6 years	1	1	1	1	1	1
Primary or less than 6 years	1.31	1.37	1.46	1.18	1.26	1.17
No formal education	1.61^a^	1.84^a^	1.92^a^	1.15	1.22	1.11
Union status^c^						
Current partnership	1	1	1	1	1	1
Single	1.28^a^	1.7^a^	2.13^a^	1.43^a^	1.38	1.47^a^
Living arrangement^d^						
Only 50 plus	1	1	1			
Skip generation	0.40^a^	0.73	0.34			
Younger adults in household	0.56^a^	0.66^a^	0.62^a^			
Employment^e^						
Not working	1	1	1			
Working	0.69^a^	0.55^a^	0.70^a^			
Nationality of origin						
South African	1	1	1			
Mozambican	1.06	0.98	1.17			
Household assets score^f^						
Highest	1	1	1	1	1	1
High	1.33	1.25	1.57	1.22	1.06	1.35
Medium	1.36	1.42	1.49	1.26	1.24	1.27
Low	1.52^a^	1.73^a^	1.65^a^	1.35	1.67^a^	1.2
Lowest	2.03^a^	1.79^a^	2.39^a^	1.71^a^	1.62	1.8^a^
Health status						
Highest	1	1	1			
High	1.16	1.14	1.32			
Medium	1.27	1.37^a^	1.39			
Low	1.70^a^	2.19^a^	1.71^a^			
Lowest	2.84^a^	3.40^a^	3.16^a^			
Work difficulty						
None	1	1	1			
Moderate	1.24	1.35	1.28			
Severe	2.39^a^	2.50^a^	2.39^a^			
WHODAS II^g^						
Best	1	1	1	1	1	1
Good	0.99	1.50	0.78	1.05	1.38	0.76
Medium	1.33	1.58	1.31	1.32	1.59	1.08
Bad	1.65^a^	2.39^a^	1.56^a^	1.54^a^	1.97^a^	1.19^a^
Worst	3.02^a^	3.85^a^	3.06^a^	2.40^a^	2.80^a^	1.92^a^
Self-reported health today						
Good	1	1	1			
Moderate	1.41^a^	1.41	1.51^a^			
Bad	2.01^a^	2.47^a^	1.83^a^			
WHOQOL^h^						
Highest	1	1	1	1	1	1
High	1.31	2.31^a^	0.88	1.16	1.64	0.81
Medium	1.21	2.08^a^	0.94	1.12	1.59	0.86
Low	1.56^a^	1.87^a^	1.54	1.14	0.98	1.13
Lowest	2.81^a^	4.03^a^	2.35^a^	1.64^a^	1.92^a^	1.41^a^

^a^Hazard ratio significantly different from 1.

^b^Number of completed years of education.

^c^Single includes never in a partnership, widowed, divorced or separated.

^d^Household structure was classified as: (i) only adults 50 years of age or older; (i) ‘skip generation’ where people 50 or older live with children under 18 and no younger adults; (iii) household which includes younger adults.

^e^Formally employed in 2004.

^f^Household measure used as a proxy for socioeconomic status.

^g^World Health Organization Disability Assessment Schedule II is a self-rated measure of functionality, presented in quintiles.

^h^World Health Organization Quality of Life score is self-rated measure of satisfaction with life, presented in quintiles.

As described in the methods section above, we constructed three multivariate Cox regression models: for the total population, for men and for women, separately (Table 3). We retained in the model all variables where the likelihood ratio test returned a value of *P* > 0.1. The survival advantage of women was maintained [HR 0.35) 95% CI 0.27–0.44)]. Age group, education status, union status, household asset score, WHOQOL and WHODAS were retained in all three models, whereas health status was discarded.

In the fully adjusted model for women, although there was no clear double peak of mortality with age, the mortality risk was similar for all ages between 50 and 79, suggesting that the younger age groups were experiencing higher mortality than would be expected. Poor physical function was associated with a greater risk of dying; those women reporting the worst function had nearly double the risk of dying compared with those in the highest function group [HR 1.92 (95% CI 1.16–3.16)]. In addition, lower socioeconomic status and not being in a current partnership were associated with a higher risk of dying independently of disability.

The fully adjusted model for men shows a clear double peak in age-related mortality, with an increased mortality risk up to age 69: 60–64, HR 2.10 (95% CI 1.00–4.39) and 65–69, HR 2.03 (95% CI 1.01–4.09). Only from age 85 onwards, the risk of dying was again higher than that in men in their 60 s.

We grouped cause of death into broad categories because there were relatively few deaths. These categories were chronic disease (164 deaths), HIV/TB (78 deaths), other infections (67 deaths), other causes (20 deaths) and unknown (48 deaths). In Table 4 we show the same general population model for each of the first three of these categories separately. The model is shown in more detail in Supplementary Table 4 (available as Supplementary data at *IJE* online). Whereas the models for chronic diseases and other infections show the expected age gradient with increasing risk at older ages, the model for HIV/TB shows an inverted relationship with age such that older people are at lower risk.

**Table 4.** Fully adjusted^a^ Cox regression analysis of risk of death of persons 50 years old or older in the Agincourt sub-district cohort (2006–09) by cause of death group, showing only hazard ratios for sex and age group

Sex and age	Chronic disease	HIV/TB	Other infections
	Hazard ratio	Hazard ratio	Hazard ratio
Male	1	1	1
Female	0.36*	0.39*	0.33*
50–54	1	1	1
55–59	1.36	0.73	0.92
60–64	1.40	0.78	1.40
65–69	2.29	0.61	1.95
70–74	1.87	0.39*	1.40
75–79	2.19*	0.19*	1.55
80–84	2.59*	1.37	1.85
85+	4.48*	0.24*	3.64*

^a^Fully adjusted model: adjusted for age, gender, union status, employment, nationality, socioeconomic status, education, mobility, self-reported health, quality of life, WHODAS and health score.

*Level of significance with *P*-value <0.05.

## Discussion

We have presented the results of 3 years of follow-up on over 4000 older people living in rural South Africa. We set out to describe some of the factors associated with mortality in a cohort of older people living in an area of rural South Africa with high HIV prevalence. One of the strengths of this study is that it is based in a sub-district that is covered by a health and demographic surveillance system, where the population has been followed for 18 years and there is access to high-quality mortality and cause of death data. Moreover, we achieved high follow-up of the participants in the study, with only 1.2% lost to follow-up at 3 years. All those recruited at baseline had been permanently resident in the research site for at least 12 months before recruitment, thus reducing the likelihood of including labour migrants who had ‘returned home to die’. There is inevitably some bias in recruitment at baseline, in that those who were unable to respond, usually because they had died or were too sick (8.1%), were not included as were those who were not present to be interviewed (28.7%). Excluding people who were ill will result in under-representation of those in poor health, whereas the exclusion of the much larger number of individuals who were not available could influence the results either positively or negatively.

For rural sub-Saharan Africa, there was a high proportion of participants living in households without younger adults, including those older persons living in ‘skip generation’ households. This might be related to the impact of the HIV epidemic on younger adults as well as the increasing number of young men and women joining the migrant work force.^29^ The situation is likely to impact on the well-being and health of the older population, although only the univariate analysis shows higher mortality in those people living in households without younger adults or children, possibly due to the weight of other socio-demographic factors such as education and socioeconomic status. Moreover, we observed the lowest mortality in the small group of individuals (2.4%) living in ‘skip generation’ households, although this might be accounted for by selection bias, in that only fitter older people become sole caregivers for children.

People of Mozambican origin have a higher risk of mortality in the univariate analysis, an effect that it is not observed in the multivariate analysis. This may be because people of South African origin suffer higher levels of HIV.^11^

Our results demonstrate higher survival in women. Individuals who were not in a partnership had lower survival, as did those with lower socioeconomic status, higher levels of disability and lower quality of life. We have previously demonstrated that women in this cohort report higher disability and lower quality of life even after adjusting for the effect of age.^12^ These findings reflect the ‘male-female health survival paradox’^30^^,^^31^ frequently observed in high-income countries, where women report poorer health and quality of life and yet have lower death rates. It may be related to some other set of health protective mechanisms, whether biologically-based or involving health-promoting behaviours or forms of social protection in families, but we are unable to investigate this further with these data.

A striking finding is the pattern of mortality, particularly in men, with an apparent fall in mortality with increasing age after an earlier rise. We do not believe that this is due to errors in recorded date of birth. Although all participants in this study were born before 1992 when the first census round was undertaken, and birthdates were not certified by the HDSS field team, those dates were later compared and corrected with those in the National ID cards that most of the population hold. Moreover, ages were further analysed looking for trends that could indicate age misreporting (for example, low numbers in a specific age group compared with others), but no evident trend in misreporting was found.

We have shown, using HDSS data from 1993 to 2010, that mortality rose dramatically in this older population (Figure 1). The effect is more pronounced for men than for women and is probably accounted for by an increasing number of deaths from HIV and TB (Figure 2). This is confirmed by comparison of the different causes of death models (Table 4). There is highest mortality in the age group 50–54 years for the model including HIV/TB deaths, but the more usual higher mortality at older ages for models including chronic diseases and other infections.

Our observations suggest that older people in the Agincourt sub-district are experiencing overall worsening of mortality. These changes are probably due to a combination of the direct effect of the HIV epidemic on older adults, and the effects of the HIV epidemic on the survival of younger adults. Whereas mortality remains higher than in the earliest time period, the most recent period—from 2007 to 2010 (Figures 1 and 2)—has seen reduction from the highest levels of mortality in both men and women. This may be related to the introduction of antiretroviral therapy in the area from 2007.

This population is continuing to age and moreover, as highly active antiretroviral therapy becomes more available, growing numbers of people living with HIV will survive to enter the older age groups. Efforts to enhance the well-being of older persons will become increasingly important. These findings have important implications for rural South African communities and for the development of health and social systems.Policy makers and practitioners should consider the needs of this growing and often overlooked group.

## Supplementary Data

Supplementary data are available at *IJE* online.

## Funding

This work was supported by the National Institute on Aging at the National Institutes of Health, USA through an interagency agreement with the World Health Organization and also through the grant number 1-P01-AG041710, the Wellcome Trust, UK (grant numbers 058893/Z/99/A and 069683/Z/02/Z), the Medical Research Council South Africa and the University of the Witwatersrand, South Africa.

## Supplementary Material

Supplementary Data
