# Supplementary material for: Social conditions and disability related to the mortality of older people in rural South Africa
Source: Int J Epidemiol. 2014 May 15;43(5):1531–41. doi: 10.1093/ije/dyu093 (PMC4190514; doi:10.1093/ije/dyu093)
Supplement: Supplementary Data [file supp_43_5_1531__index.html]

Social conditions and disability related to the mortality of older people in rural South Africa — Social conditions and disability related to the mortality of older people in rural South Africa — Supplementary Data 

## Supplementary Data

files

**Files in this Data Supplement:**

- Supplementary Data - docx file
