# Supplementary material for: Social conditions and disability related to the mortality of older people in rural South Africa
Source: Int J Epidemiol. 2014 May 15;43(5):1531–41. doi: 10.1093/ije/dyu093 (PMC4190514; doi:10.1093/ije/dyu093)
Supplement: Supplementary Data [file supp_dyu093_ije-2012-02-0137-File004.docx]

**WEB ANNEX**

## Web Table 1 –Death rates per 1000 person years in study participants and in the whole Agincourt research site population aged 50 years and older by gender.

| Age group* | Male SAGE  2006-2009  (95% CI) | Male census 2003-2006  (95% CI) | Male census  2007-2010  (95% CI) | Female SAGE  2006-2009  (95% CI) | Female census  2003-2006  (95% CI) | Female census 2007-2010  (95% CI) |
| --- | --- | --- | --- | --- | --- | --- |
| 50-54 | 31.4 (17.4-56.8) | 69.3 (57.6-83.3) | 58.0 (48.3-69.7) | 20.3 (14.1-29.2) | 26.1 (21.2-32.0) | 21.7 (17.8-26.5) |
| 55-59 | 42.0 (25.7-68.6) | 74.9 (61.4-91.4) | 53.2 (43.9-64.4) | 18.3 (12.3-27.0) | 21.5 (16.8-27.7) | 23.1 (18.7-28.7) |
| 60-64 | 74.2 (48.9-112.7) | 70.7 (57.7-86.5) | 65.5 (53.6-80.1) | 14.8 (9.5-23.3) | 20.9 (16.1-27.1) | 19.9 (15.5-25.7) |
| 65-69 | 63.6 (45.4-89.0) | 59.0 (47.8-72.8) | 53.0 (42.9-65.6) | 30.4 (21.9-42.1) | 21.7 (16.7-28.3) | 23.8 (18.9-30.1) |
| 70-74 | 53.8 (34.3-84.3) | 65.3 (50.4-84.6) | 62.8 (50.1-78.7) | 25.0 (17.2-36.5) | 28.0 (22.1-35.4) | 32.1 (25.6-40.1) |
| 75-79 | 49.6 (31.2-78.7) | 77.1 (59.9-99.4) | 78.4 (61.5-100.0) | 32.3 (23.3-44.8) | 34.2 (27.0-43.3) | 34.2 (27.6-42.2) |
| 80-84^$^ | 64.1 (34.5-119) | 117.3 (90.9-151.4) | 98.7 (75.0-129.9) | 40.2 (25.9-62.3) | 63.9 (49.2-83.1) | 56.2 (45.6-69.3) |
| 85+ | 128.2 (87.3-188.2) |  |  | 76.9 (53.5-110.7) |  |  |

*Ages at the start point of recruitment in August 2006.

^$ In census columns this age group represents 80 plus^

## Web Table 2 Univariate Cox regression analysis of risk of death of persons 50 years old or older in the Agincourt sub-district cohort (2006 – 2009) by gender.

|  | **Total** | **Male** | **Female** |
| --- | --- | --- | --- |
|  | **Hazard ratio (95% CI)** | **Hazard ratio (95% CI)** | **Hazard ratio (95% CI)** |
| **Sex** |  |  |  |
| Male | 1 |  |  |
| Female | **0.45 (0.36 - 0.55)** |  |  |
| **Age group** |  |  |  |
| 50 - 54 | 1 | 1 | 1 |
| 55-59 | 1.04 (0.7 - 1.6) | 1.33 (0.62 - 2.87) | 0.90 (0.53 - 1.54) |
| 60-64 | 1.16 (0.7 - 1.8) | **2.37 (1.15 - 4.88)** | 0.73 (0.41 - 1.30) |
| 65-69 | **1.81 (1.2 - 2.7)** | 2.02 (1.02 - 3.98) | 1.50 (0.92 - 2.45) |
| 70-74 | 1.43 (0.9 - 2.2) | 1.7 (0.81 - 3.57) | 1.23 (0.73 - 2.09) |
| 75-79 | **1.63 (1.1 - 2.4)** | 1.57 (0.74 - 3.32) | 1.59 (0.98 - 2.60) |
| 80-84 | **2.04 (1.3 - 3.3)** | 2.02 (0.86 - 4.76) | **1.99 (1.12 - 3.52)** |
| 85+ | **4.27 (2.8 – 6.4)** | **4.11 (2.03 - 8.32)** | **3.84 (2.29 – 6.42)** |
| **Education Status*** |  |  |  |
| More than 6 years | 1 | 1 | 1 |
| Primary or less than 6 years | 1.31 (0.88 - 1.96) | 1.37 (0.79 - 2.38) | 1.46 (0.81 - 2.66) |
| No formal education | **1.61 (1.14 - 2.26)** | **1.84 (1.16 - 2.91)** | **1.92 (1.15 - 3.21)** |
| **Union status**** |  |  |  |
| Current partnership | 1 | 1 | 1 |
| Single | **1.28 (1.04 - 1.57)** | **1.7 (1.21 - 2.37)** | **2.13 (1.54 - 2.94)** |
| **Living arrangement^&^** | |  |  |
| Only 50 plus | 1 | 1 | 1 |
| Skip generation | **0.40 (0.17 - 0.92)** | 0.73 (0.22 - 2.39) | 0.34 (0.10 - 1.14) |
| Younger adults in hh | **0.56 (0.42 - 0.74)** | **0.66 (0.45 - 0.97)** | **0.62 (0.41 - 0.94)** |
| **Employment^&&^** |  |  |  |
| Not working | 1 | 1 | 1 |
| Working | **0.69 (0.49 - 0.96)** | **0.55 (0.34 - 0.90)** | **0.70 (0.44 - 1.10)** |
| **Nationality of origin** | |  |  |
| South African | 1 | 1 | 1 |
| Mozambican | 1.06 (0.84 - 1.32) | 0.98 (0.68 - 1.42) | 1.17 (0.88 - 1.55) |
| **Household assets score^#^** | |  |  |
| Highest | 1 | 1 | 1 |
| High | 1.33 (0.96 - 1.84) | 1.25 (0.76 - 2.05) | **1.57 (1.01 - 2.43)** |
| Medium | 1.36 (0.97 - 1.90) | 1.42 (0.86 - 2.32) | 1.49 (0.94 - 2.36) |
| Low | **1.52 (1.09 - 2.12)** | **1.73 (1.05 - 2.86)** | **1.65 (1.05 - 2.60)** |
| Lowest | **2.03 (1.46 - 2.81)** | **1.79 (1.09 - 2.92)** | **2.39 (1.53 - 3.73)** |
| **Health Status** |  |  |  |
| Highest | 1 | 1 | 1 |
| High | 1.16 (0.81 - 1.68) | 1.14 (0.67 - 1.96) | 1.32 (0.80 - 2.18) |
| Medium | 1.27 (0.88 - 1.84) | **1.37 (0.79 - 2.37)** | 1.39 (0.83 - 2.31) |
| Low | **1.70 (1.20 - 2.40)** | **2.19 (1.34 - 3.60)** | **1.71 (1.05 - 2.76)** |
| Lowest | **2.84 (2.07 - 3.88)** | **3.40 (2.14 - 5.42)** | **3.16 (2.05 - 4.89)** |
| **Work difficulty** |  |  |  |
| None | 1 | 1 | 1 |
| Moderate | 1.24 (0.98 - 1.58) | 1.35 (0.92 - 1.96) | 1.28 (0.93 - 1.74) |
| Severe | **2.39 (1.86 - 3.07)** | **2.50 (1.71 - 3.67)** | **2.39 (1.72 - 3.32)** |
| **WHODAS II^$^** |  |  |  |
| Best | 1 | 1 | 1 |
| Good | 0.99 (0.67 - 1.46) | 1.50 (0.87 - 2.59) | 0.78 (0.45 - 1.35) |
| Medium | 1.33 (0.92 - 1.91) | 1.58 (0.91 - 2.75) | 1.31 (0.81 - 2.13) |
| Bad | **1.65 (1.18 - 2.31)** | **2.39 (1.44 - 3.97)** | **1.56 (1.00 - 2.44)** |
| Worst | **3.02 (2.24 - 4.07)** | **3.85 (2.44 - 6.07)** | **3.06 (2.05 - 4.56)** |
| **Self Reported Health today** | |  |  |
| Good | 1 | 1 | 1 |
| Moderate | **1.41 (1.11 - 1.77)** | 1.41 (0.97 - 2.05) | **1.51 (1.12 - 2.04)** |
| Bad | **2.01 (1.55 - 2.61)** | **2.47 (1.68 - 3.63)** | **1.83 (1.29 - 2.61)** |
| **WHOQOL^$$^** |  |  |  |
| Highest | 1 | 1 | 1 |
| High | 1.31 (0.91 - 1.90) | **2.31 (1.31 - 4.07)** | 0.88 (0.54 - 1.46) |
| Medium | 1.21 (0.82 - 1.77) | **2.08 (1.14 - 3.81)** | 0.94 (0.57 - 1.55) |
| Low | **1.56 (1.09 - 2.23)** | **1.87 (1.00 - 3.48)** | 1.54 (0.99 - 2.39) |
| Lowest | **2.81 (2.02 - 3.90)** | **4.03 (2.37 - 6.87)** | **2.35 (1.54 - 3.58)** |

**Values in bold show significant effect.**

*****“Education status” refers to the number of completed years of education.

******“Union status”: In current partnership refers to those in a union; Single includes never in a union, widowed, divorced or separated.

**&** “Living arrangement” Household structure was divided in three categories: i) Only adults 50 years or older; ii) “Skip generation” household is defined as a household were people older than 50 lives with children under 18 years of age and there are no persons 18 to 49 years of age; iii) “Younger adults” defined as presence of both persons older and younger than 50 years in the household.

**&&** “Employment” refers to being formally employed in 2004.

**#** “Household asset score” household weighted measured used as a proxy to calculate socio-economic status.

**$** “WHODAS II” (World Health Organisation Disability Assessment Schedule II ) is a self rated measure of functionality presented in quintiles.

**$$** “WHOQOL” (World Health Organisation quality of life) is a self-rated overall satisfaction with life presented in quintiles

## Web Table 3. Fully adjusted^†^ Cox regression analysis of risk of death of persons 50 years old or older in the Agincourt sub-district cohort (2006 – 2009) by gender.

|  | **General** | **Male** | **Female** |
| --- | --- | --- | --- |
| **Gender** | **Hazard ratio (95% CI)** | **Hazard ratio (95% CI)** | **Hazard ratio (95% CI)** |
| Male | 1 |  |  |
| Female | **0.35 (0.27 - 0.44)** |  |  |
| **Age group** |  |  |  |
| 50 - 54 | 1 | 1 | 1 |
| 55-59 | 0.9 (0.57 - 1.43) | 1.14 (0.52 - 2.52) | 0.81 (0.46 - 1.45) |
| 60-64 | 1.11 (0.70 - 1.75) | **2.10 (1.00 - 4.39)** | 0.71 (0.38 - 1.32) |
| 65-69 | 1.44 (0.95 - 2.18) | **2.03 (1.01 - 4.09)** | 1.2 (0.7 - 2.06) |
| 70-74 | 1.01 (0.64 - 1.58) | 1.33 (0.62 - 2.89) | 0.95 (0.54 - 1.67) |
| 75-79 | 1.06 (0.68 - 1.66) | 1.23 (0.55 - 2.72) | 1.06 (0.61 - 1.85) |
| 80-84 | 1.30 (0.78 - 2.15) | 1.26 (0.50 - 3.16) | 1.37 (0.73 - 2.55) |
| 85+ | **2.27 (1.44 - 3.58)** | **3.03 (1.45 - 6.32)** | **2.07 (1.13 - 3.79)** |
| **Education Status^*^** |  |  |  |
| More than 6 years | 1 | 1 | 1 |
| Primary or less than 6 years | 1.18 (0.78 - 1.78) | 1.26 (0.72 - 2.21) | 1.17 (0.63 - 2.16) |
| No formal education | 1.15 (0.79 - 1.66) | 1.22 (0.75 - 2.01) | 1.11 (0.64 - 1.92) |
| **Union status^**^** |  |  |  |
| Current partnership | 1 | 1 | 1 |
| Single | **1.43 (1.11 - 1.84)** | 1.38 (0.95 - 2.00) | **1.47 (1.02 - 2.1)** |
| **Household assets score^#^** |  |  |  |
| Highest | 1 | 1 | 1 |
| High | 1.22 (0.87 - 1.72) | 1.06 (0.63 - 1.79) | 1.35 (0.85 - 2.14) |
| Medium | 1.26 (0.89 - 1.78) | 1.24 (0.75 - 2.07) | 1.27 (0.79 - 2.05) |
| Low | 1.35 (0.95 - 1.92) | **1.67 (1.00 - 2.80)** | 1.2 (0.74 - 1.94) |
| Lowest | **1.71 (1.20 - 2.43)** | 1.62 (0.96 - 2.72) | **1.8 (1.11 - 2.93)** |
|  |  |  |  |
|  |  |  |  |
|  |  |  |  |
| **WHODAS^$^** |  |  |  |
| Best | 1 | 1 | 1 |
| Good | 1.05 (0.60 - 1.58) | 1.38 (0.76 - 2.47) | 0.76 (0.43 - 1.37) |
| Medium | 1.32 (0.89 - 1.96) | 1.59 (0.89 - 2.85) | 1.08 (0.63 - 1.85) |
| Bad | **1.54 (1.06 - 2.25)** | **1.97 (1.12 - 3.47)** | **1.19 (0.72 - 1.97)** |
| Worst | **2.40 (1.65 - 3.47)** | **2.80 (1.60 - 4.89)** | **1.92 (1.16 - 3.16)** |
| **WHOQOL^$$^** |  |  |  |
| Highest | 1 | 1 | 1 |
| High | 1.16 (0.78 - 1.72) | 1.64 (0.90 - 2.96) | 0.81 (0.47 - 1.4) |
| Medium | 1.12 (0.74 - 1.70) | 1.59 (0.85 - 3.00) | 0.86 (0.49 - 1.48) |
| Low | 1.14 (0.75 - 1.71) | 0.98 (0.49 - 1.96) | 1.13 (0.68 - 1.89) |
| Lowest | **1.64 (1.11 - 2.44)** | **1.92 (1.03 - 3.57)** | 1.41 (0.84 - 2.38) |

**Values in bold show significant effect.**

**^†^** Fully adjusted model: adjusted for age, gender, union status, employment, nationality, socio-economic status, education, mobility, self reported health, quality of life, WHODAS and health score.

***** “Education status” refers to the number of completed years of education.

****** “Union status”: In current partnership refers to those in a union; Single includes never in a union, widowed, divorced or separated.

**#** “Household asset score” household weighted measured used as a proxy to calculate socio-economic status.

**$** “WHODAS II” (World Health Organisation Disability Assessment Schedule II ) is a self rated measure of functionality presented in quintiles.

**$$** “WHOQOL” (World Health Organisation quality of life) is a self-rated overall satisfaction with life presented in quintiles

## Web Table 4. Fully adjusted† Cox regression analysis of risk of death of persons 50 years old or older in the Agincourt sub-district cohort (2006 – 2009) by cause of death group.

|  | **Chronic Disease** | **HIV/TB** | **Other infections** |
| --- | --- | --- | --- |
| **Gender** | **Hazard ratio (95% CI)** | **Hazard ratio (95% CI)** | **Hazard ratio (95% CI)** |
| Male | 1 | 1 | 1 |
| Female | **0.36 (0.25 - 0.52)** | **0.39 (0.24 - 0.65)** | **0.33 (0.19 - 0.58)** |
| **Age group** |  |  |  |
| 50 - 54 | 1 | 1 | 1 |
| 55-59 | 1.36 (0.63 - 2.92) | 0.73 (0.36 - 1.46) | 0.92 (0.27 - 3.19) |
| 60-64 | 1.40 (0.64 - 3.10) | 0.78 (0.38 - 1.60) | 1.40 (0.43 - 4.62) |
| 65-69 | 2.29 (1.14 - 4.62) | 0.61 (0.30 - 1.26) | 1.95 (0.68 - 5.59) |
| 70-74 | 1.87 (0.89 - 3.92) | **0.39 (0.16 - 0.91)** | 1.40 (0.46 - 4.29) |
| 75-79 | **2.19 (1.07 - 4.49)** | **0.19 (0.06 - 0.57)** | 1.55 (0.51 - 4.64) |
| 80-84 | **2.59 (1.17 - 5.73)** | 1.37 (0.12 - 1.12) | 1.85 (0.55 - 6.17) |
| 85+ | **4.48 (2.15 - 9.32)** | **0.24 (0.07 - 0.87)** | **3.64 (1.24 - 10.70)** |
| **Education Status^*^** |  |  |  |
| More than 6 years | 1 | 1 | 1 |
| Primary or less than 6 years | 1.14 (0.59 - 2.21) | 0.88 (0.43 - 1.79) | 1.91 (0.61 - 6.00) |
| No formal education | 1.37 (0.77 - 2.41) | 0.73 (0.39 - 1.37) | 1.51 (0.52 - 4.39) |
| **Union status^**^** |  |  |  |
| Current partnership | 1 | 1 | 1 |
| Single | 1.20 (0.83 - 1.74) | **2.27 (1.35 - 3.82)** | **2.32 (1.26 - 4.28)** |
| **Household assets score^#^** |  |  |  |
| Highest | 1 | 1 | 1 |
| High | 0.98 (0.60 - 1.60) | **2.19 (1.00 - 4.77)** | 1.59 (0.74 - 3.41) |
| Medium | 1.22 (0.75 - 1.98) | 1.66 (0.73 - 3.78) | 1.14 (0.49 - 2.65) |
| Low | 1.17 (0.70 - 1.93) | 2.02 (0.90 - 4.55) | 1.08 (0.46 - 2.54) |
| Lowest | 1.31 (0.79 - 2.17) | **2.60 (1.14 - 5.92)** | 1.78 (0.79 - 4.02) |
| **WHOQOL^$$^** |  |  |  |
| Highest | 1 | 1 | 1 |
| High | 1.50 (0.85 - 2.63) | 1.76 (0.78 - 3.96) | 0.69 (0.25 - 1.92) |
| Medium | 1.27 (0.70 - 2.30) | 1.10 (0.44 - 2.76) | 0.85 (0.32 - 2.28) |
| Low | 1.03 (0.56 - 1.91) | 1.26 (0.52 - 3.06) | 1.10 (0.45 - 2.71) |
| Lowest | **1.81 (1.02 - 3.21)** | 1.99 (0.85 - 4.66) | 1.30 (0.53 - 3.18) |
| **WHODAS^$^** |  |  |  |
| Best | 1 | 1 | 1 |
| Good | 0.77 (0.41 - 1.43) | 0.88 (0.40 - 1.94) | 2.53 (0.73 - 8.72) |
| Medium | 1.21 (0.70 - 2.10) | 1.19 (0.55 - 2.56) | 3.16 (0.94 - 10.55) |
| Bad | 1.61 (0.97 - 2.67) | 1.00 (0.46 - 2.17) | **5.29 (1.70 - 16.47)** |
| Worst | **1.77 (1.05 - 3.01)** | **2.38 (1.15 - 4.93)** | **6.55 (2.07 - 20.74)** |

**Values in bold show significant effect.**

**^†^** Fully adjusted model: adjusted for age, gender, union status, employment, nationality, socio-economic status, education, mobility, self reported health, quality of life, WHODAS and health score.

***** “Education status” refers to the number of completed years of education.

****** “Union status”: In current partnership refers to those in a union; Single includes never in a union, widowed, divorced or separated.

**#** “Household asset score” household weighted measured used as a proxy to calculate socio-economic status.

**$** “WHODAS II” (World Health Organisation Disability Assessment Schedule II ) is a self rated measure of functionality presented in quintiles.

**$$** “WHOQOL” (World Health Organisation quality of life) is a self-rated overall satisfaction with life presented in quintiles
